# Supplementary material for: Can we predict the severe course of COVID-19 - a systematic review and meta-analysis of indicators of clinical outcome?
Source: PLoS One. 2021 Jul 29;16(7):e0255154. doi: 10.1371/journal.pone.0255154 (PMC8321230; doi:10.1371/journal.pone.0255154)
Supplement: Attachment — Submitted filename: Rebuttal Letter PLOS One.docx [file pone.0255154.s003.docx]

Rebuttal Letter

„Can we predict the severe course of COVID-19 – a systematic review and meta-analysis of indicators of clinical outcome?”

| **Reviewer #1** | **Response from Authors** |
| --- | --- |
| **Page 5** The search period runs up to the time when dexamethasone first became recommended. Should this be made explicit? Is it deliberate? | Thank you for the suggestion. The RECOVERY trial was published on July 17th with an inclusion period up until June 8th, our search period runs up to May 31st. Dexamethasone (or hydrocortisone) was probably used more frequently in critically ill patients during the end of our search period. However, it was not the goal of this systematic review and meta-analysis to identify therapies that are correlated with a worse outcome. Instead, we focused on clinical parameters the treating physician would have available within the first hours to identify patients at higher risk for mortality and/or ICU admission. |
| **Page 7** The idea of meta–analysing medians may not be familiar to readers as it has been relatively recently made available. I wonder whether some more explanation might be added here specifically about the slightly different interpretations compared to meta-analysis of means. These are explained in the paper the authors cite (McGrath et al., 2020). | Thank you for the suggestion. We added a statement in the “Statistical analysis” subsection of the manuscript to clarify the interpretation of the pooled estimate of the difference of medians. |
| **Page 7** REML failing to converge is unusual. Given it is rare it is perhaps not serious but I note that the same biomarkers are implicated most of the time for the different outcomes so have the authors any insights from the distributions involved? | First, we would like to clarify when the REML algorithm failed to converge. REML only failed to converge when pooling the median DDimer value in the non-ICU group. For the other two outcomes in which we note the REML algorithm failed to converge (i.e., Troponin-I and CRP), we received a warning message originating from the *meta for* R package stating that the ratio of largest sampling variance to the smallest sampling variance is very large, although the algorithm did not fail to converge in a strict sense. For consistency, whenever we switched to using the DerSimmonian and Laird method to estimate heterogeneity for an outcome, we used this method in all analyses for that outcome.  In the analysis where REML failed to converge, there were three large outlier studies (study specific estimates greater than 400) compared to the other 11 studies (study-specific estimates between 0 and 12) and there was a large ratio of largest sampling variance to the smallest sampling variance.  In the revised manuscript, we clarified in the “Statistical analysis” subsection when we used the DerSimonian and Laird method instead of REML. |
| **Page 7** What funnel plots attempt to show us are small study effects. Call- ing it publication bias implies you know the aetiology. | We agree and replaced both instances of the phrase “publication bias” in the manuscript with “small-study effects”. |
| **Page 8** Are the indicators presented the totality of those for which the authors planned to extract data? If there were some which were planned but not achieved we should know that. The supplement reveals there were other variables extracted so an explicit statement about exclusion would help. Are these all covered by the comment in the methods on page 6? | Data for some indicators were insufficient for meta-analysis. We listed all the indicators we extracted in the Supplements (S12). We mentioned S12 already on page 6, but are happy to accentuate that not every indicator was sufficient for a meta-analysis. A list of the indicators that we meta-analysed can be found under S5 to S8. We provided a clear statement on page 8. |
| **Page 9** Here and subsequently the authors report both clinical and statisti- cal significance. I am all for this, statistical significance is over–valued, but how did they decide on the limits of clinical significance? | Clinical significance was achieved through expert consent with clinicians. We state this in the method section. A predefined rule across biomarkers is not possible as it is different for every biomarker and the unit chosen. E.g. a doubling of CRP from normal is not clinically significant, while for troponin it is |
| **Figure 1** In the methods we learn that there was no language exclusion but here we find studies excluded as ”other language”. | There are language exclusions, we edited this mistake in the methods section on Page 5. |
| **Figure 4** I do not see anything here in grey. If the bars are supposed to represent the normal range then I do not understand the comment in the caption about the upper limit of leukocytes and lymphocytes as the bars do not extend to 1×10^10^ and 4×10^9^ respectively. | We apologize for this mistake. The grey shading in this figure appears to have been removed during the file conversion. We have changed the file type so that this does not occur again. |
| **Figure S12** For the record presenting the results with and without the Yang study is informative even though most people might have just excluded Yang altogether. | We appreciate the comment but decided to leave it in. |
| **Figure S17** and subsequently Is θi = θj a standard way of expressing this? I do not think I have seen it before. θ = 0 seems more standard. | In Stata’s meta command, the notation θ_i_=θ_j_ is the default output to express the null hypothesis of the test of homogeneity (Cochrane’s Q) and θ=0 is the default output to express the null hypothesis of the test of the overall effect size. We would be happy to revise the notation if the reviewer finds it distracting. |
| **Funnel plots** If the lines are supposed to be 95% significance values then the vertical line should be at the null value not the estimate. Some of these have rather few studies and I might have excluded the funnel plots but I suppose there is no harm in presenting them. | The dotted lines indeed represent the pseudo 95% confidence limits. Centering the funnel plot at the estimate compared to the null value is common practice to the best of our knowledge. Further, we believe that (i) the interpretation of the confidence limits would no longer be very meaningful when centering at the null value and (ii) readers may mistakenly believe there is substantial asymmetry in the funnel plots when centering at the null.  If the reviewer was simply requesting us to add a vertical line at the null value in the funnel plots, we would be happy to do so.  In the revised manuscript, we indicated that the dotted lines represent the pseudo 95% confidence limits. |

| **Reviewer #2** | **Response from Authors** |
| --- | --- |
| This is a clear and comprehensive review of published data (peer-reviewed and preprint) that assesses the risk factors for ICU admission and mortality in COVID-19. The literature search has been well conducted and the methods of aggregating data are appropriate. | Thank you for your comment. |
| The difficulty is that there are now many published systematic reviews of this topic, and it is not clear what this manuscript adds. Findings from this systematic review are similar to what is reported in other reviews (e.g. <https://journals.plos.org/plosone/article?id=10.1371/journal.pone.0241742>) and while the pooling of medians for laboratory values is less commonly performed and preferable to binary 'normal/abnormal', this too is not unique (<https://onlinelibrary.wiley.com/doi/10.1002/jmv.26050>). | Regarding the study you are citing we are adding risk factors for ICU admission with the same methodology as we did for mortality. Therefore this meta-analysis can help clinicians evaluate which patient is at risk for ICU admission, as we are now facing capacity limits again all over the world. Furthermore, we analyzed the risk factors by hospital admission and intubation in addition to ICU admission and mortality. This allowed us to further cross validate the analysis.  In addition, the analysis by median allowed us to combine substantially more data from papers. |
| The authors note there is considerable bias and heterogeneity in the data - but this makes it difficult to draw robust conclusions as the authors try to do. For example, the authors reference the large study (Williamson et al) from the UK using the OpenSAFELY data where hypertension was not a risk factor for COVID-19 mortality. However, on further analysis Williamson et al. identified 'strong evidence of interaction (P < 0.001), with hypertension associated with a higher risk up to the age of 70 years and a lower risk above the age of 70'. While the explanation for this is not clear (and it does require confirmation), with the quantity of data already available what is really needed is an individual patient data meta-analysis which can uncover deeper insights rather than further pooling of study level data. | Unfortunately, we are unable to perform age-related analysis (meta-regression) as the necessary data is not presented in the papers included. Therefore we cannot make a statement whether an indicator is a risk factor for a certain age group or not.  We agree with the opinion of the reviewer on the need for individual patient data meta-analysis. |

| **Reviewer #3** | **Response from Authors** |
| --- | --- |
| Many thanks for the opportunity to review the manuscript of Katzenschlager et al.  As the title "Can we predict the severe course of COVID-19 - a systematic review and meta-analysis of indicators of clinical outcome?" intimates this is an extensive study on the risk factors and prognostic factors of COVID19. I believe the authors completed a huge task, and I can only compliment on their study. However, none of the results in this study is new. These results replicate what we already knew from very early high-quality systematic reviews and meta-analyses in this subject. Also, due to the extensive analyses and results, the reader struggles to understand what the real focus is.  In summary, although the study has many virtues, I do not recommend it for publication in Plos Medicine. | Thank you, we appreciate your opinion and are keen to accept the opportunity to publish in PLOS One. |
