# Supplementary material for: Can we predict the severe course of COVID-19 - a systematic review and meta-analysis of indicators of clinical outcome?
Source: PLoS One. 2021 Jul 29;16(7):e0255154. doi: 10.1371/journal.pone.0255154 (PMC8321230; doi:10.1371/journal.pone.0255154)
Supplement: Attachment — Submitted filename: Rebuttal Letter PLOS One_R2_V1.docx [file pone.0255154.s004.docx]

Thank you for the possibility to respond to the editors and reviewers comments. Please note that all pages and lines refer to the unmarked manuscript.

Editorial Comment:

| Please review your reference list to ensure that it is complete and correct. If you have cited papers that have been retracted, please include the rationale for doing so in the manuscript text, or remove these references and replace them with relevant current references. Any changes to the reference list should be mentioned in the rebuttal letter that accompanies your revised manuscript. If you need to cite a retracted article, indicate the article’s retracted status in the References list and also include a citation and full reference for the retraction notice. | We have reviewed our reference list and ensured that it is complete and all papers that were published in peer reviewed journals are now listed as such. |
| --- | --- |
| Please ensure that your manuscript meets PLOS ONE's style requirements, including those for file naming. The PLOS ONE style templates can be found at  <https://journals.plos.org/plosone/s/file?id=wjVg/>  PLOSOne_formatting_sample_main_body.pdf and  <https://journals.plos.org/plosone/s/file?id=ba62/>  PLOSOne_formatting_sample_title_authors_affiliations.pdf |  |
| Please include the date(s) on which your research team performed the searches. | This is mentioned under **Methods, Eligibility criteria, Page 5, Lines 85 to 86** |
| Please remove any funding-related text from the manuscript and let us know how you would like to update your Funding Statement. Currently, your Funding Statement reads as follows:   [The study was supported by internal funds of the Heidelberg University Hospital. The funders play no role in the study design, data collection and analysis, decision to publish, or preparation of the manuscript.]  Please include your amended statements within your cover letter; we will change the online submission form on your behalf. | We have removed the funding-related text from the manuscript. |
| We note that you have stated that you will provide repository information for your data at acceptance. Should your manuscript be accepted for publication, we will hold it until you provide the relevant accession numbers or DOIs necessary to access your data. If you wish to make changes to your Data Availability statement, please describe these changes in your cover letter and we will update your Data Availability statement to reflect the information you provide. | Our data is available under XXX. We will make it publicly available once the paper is published. |
| Please include a copy of Table 3 which you refer to in your text on page 10. | We apologize for the oversight. This was mistaken with Fig. 3b and is now corrected. |

Reviewer – Overall comments

| The risk of bias evaluation was conducted and reported at the study level for a study that is focus on the evidence regarding individual risk factor-outcome level across different studies. There lies a limitation in the sense that it is hard to know what is the confidence/uncertainty surrounding each risk factor-outcome association. | Thank you for this comment. This goes in hand with your comment on “Assessment of study quality”. We have added your suggestion to this point in the **limitation** section of our paper. **Pages 17, Lines 344 to 346.**  “*However, an analysis of quality was performed at the study level. Conceivably a high-quality study will contribute more high quality data on the risk-factor outcome association, but this cannot be ascertained.”* |
| --- | --- |
| The categorical variables were presented in terms of odds ratios instead of relative risks. Odds ratios are a valid measure of epidemiological association, however there might be a risk of overestimating estimates relative to estimates calculated in terms of relative risks. I think it is worth commenting on the likelihood of this overestimation based on the authors knowledge of the literature and if there was a particular rationale supporting the use of odds ratios. | We thank the reviewer for this comment. In this paper, we are interested in the chance of being admitted to the ICU among those with fever vs no fever (as an example). To determine this, it is more logical to use the odds ratio than the relative risk. That is, what are the odds of being admitted to the ICU given the person has a fever relative to people without a fever. To compute the relative risk of ICU admission, we would need to calculate the risk of ICU among people with fever compared to the risk of ICU among people without a fever. Data that would inform relative risk estimate is more limited.  Of note, the vast majority of other systematic reviews/meta-analyses on COVID-19 risk factors have used the OR instead of RR:   - https://www.ncbi.nlm.nih.gov/pmc/articles/PMC7980087/ - https://apm.amegroups.com/article/view/61859/html - https://link.springer.com/article/10.1007/s10654-021-00732-w - Risk factors of critical & mortal COVID-19 cases: A systematic literature review and meta-analysis. Zheng Z, Peng F, Xu B, Zhao J, Liu H, Peng J, Li Q, Jiang C, Zhou Y, Liu S, Ye C, Zhang P, Xing Y, Guo H, Tang W.J Infect. 2020 Aug;81(2):e16-e25. doi: 10.1016/j.jinf.2020.04.021. Epub 2020 Apr 23.   We have acknowledged the above in the **Discussion**, **Page 15, Lines 304 to 310.**  “*For categorical variables, OR was used to measure the association between the outcome (mortality, ICU admission, hospitalization, intubation) and the risk factor/biomarker of interest. We decided to use the OR instead of the relative risk (RR) given the nature of the research question and the data that was available to us. For example, calculating the risk of ICU among people with fever compared to the risk of ICU among people without a fever is not as informative as computing the odds ratio of ICU admission between fever and non-fever patients. We acknowledge, however, when interpreting the results that the ORs are more extreme (further from the null) than the RRs whenever there is a non-null association.”* |
| In light of the above presented ideas, I would suggest changing any comments affirming that the study “confirms” most of the proposed indicators. My suggestion comes from the idea that in English language the term confirm seem to be more associated with establishing the truth or correctness of something, similar to beweisen (in German). Perhaps the authors refer confirm as bestätigen (in German) to convey validate, affirm, reaffirm, certify. It is a different claim to suggest findings support previous estimates, than to present them as an established scientific truth, especially without conducting an evidence quality analysis at the outcome level. | We thank you for this comment. We have changed the wording to “corroborate”, “identify” and “found” accordingly. |

Reviewer – Abstract

| -Methods: I would suggest reporting the PROSPERO registration in the methods section of the abstract. | We have reported the PROSPERO ID at Page 3 |
| --- | --- |
| -Results: I would suggest to include P values in abstract | Thank you for pointing out this aspect. However, based on recent guidance (Rothmann Eur J Epidemiology 2016, Amrheim Commentary Nature 2019), we would like to avoid using p-values. |
| - Discussion: By decision analytical tools are you referring to development of clinical scores for predicting outcomes? | We have adapted the paragraph. By decision analytical tools we are referring to clinical scores for early decision .  “*This comprehensive meta-analysis found age, cerebrovascular disease, CRP, LDH and cTnI to be the most important risk-factors in predicting severe COVID-19 outcomes and will inform clinical scores to support early decision-making.”* |

Reviewer – Introduction

| Perhaps add that it was October 31 2020 or October 31 of the same year. It seems redundant but it reads weirdly for me with just the month and the day. As a reader, I got distracted thinking about it. | We have added the respective year. Thank you for this comment. |
| --- | --- |
| Acknowledge the existence of previous reviews and systematic reviews discussing risk factors for covid-19 severity and highlight what is the added value of the present review:  e.g.  Risk factors of critical & mortal COVID-19 cases: A systematic literature review and meta-analysis. Zheng Z, Peng F, Xu B, Zhao J, Liu H, Peng J, Li Q, Jiang C, Zhou Y, Liu S, Ye C, Zhang P, Xing Y, Guo H, Tang W.J Infect. 2020 Aug;81(2):e16-e25. doi: 10.1016/j.jinf.2020.04.021. Epub 2020 Apr 23.  A brief-review of the risk factors for covid-19 severity JE Rod, O Oviedo-Trespalacios, J Cortes-Ramirez Rev Saude Publica. 54 (60) | Thank you for this suggestion. We had already cited the review by Zheng et al and acknowledged other reviews as well now in our introduction and discussion. |

Reviewer – Methods

| -Eligibility criteria: Please provide a brief comment on the rationale for focusing on the presented outcomes for severity e.g.: why other markers of severity as O2 saturation or the development of acute respiratory distress syndrome where not included. | Thank you for this comment. We added a short statement for the rationale on the chosen parameters at **Page 5, Lines 87 to 89**  “*A full list of data items screened for in the studies is available in the supplementary file S12. These data items were chosen, on the one hand, according to the information available in the existing literature and, on the other hand, in order to identify risk factors at hospital admission.”*  In addition we have included O2 saturation in our meta-analysis.  As this vital parameter will be measured early at admission, we have moved the respective result up to the beginning of the mortality section at **page 13, lines 238 to 241.**  “*Regarding vital parameters at admission, patients who died presented with a median peripheral oxygen saturation (SpO2) on room air of 89% (CI 87.32 to 90.91) to the hospital, while those who survived had 95% (CI 94.59 to 96.63) (DoM -6.33%, CI -8.14 to -4.52).”*  We have not analyzed ARDS as this was not reported throughout. Furthermore this review focused on initial symptoms, vital parameters and laboratory values. |
| --- | --- |
| -Study and screening: “Article title and abstracts were screened for eligibility in (instead of and?) English and German”. | We have corrected this typo. |
| -Assessment of study quality: Study quality might be a more important step in aiming at ranking the studies in a way that could perhaps exclude some studies and include only studies with high quality rankings. Here it seems that the intention was to provide a comment on the risk of bias of each study. I mention this given that the individual evaluation of the risk of bias for different outcomes (hospital admission, intubation. etc) across different papers does not necessarily share the same risk of bias as the whole paper. Eg. some papers report bivariate analysis for one set of outcomes but multivariate analysis for other outcomes.  I understand that conducting a risk of bias for each of the outcomes is extremely labor some. **However, it must be acknowledged that the risk of bias assessment was performed at the study and not the outcome level and therefor the risk of bias assessment might work as an index of a risk of bias at the outcome level, but deeper analyses might yield a different result**. | Please see our comment above on your overall comments. |
| Statistical analysis: Perhaps, but not necessarily, it would be clearer to separate study outcomes in “primary” and “secondary” based on data availability and said that secondary outcomes comparisons can be found in the supplement. This could also be reflected on the results section. | Thank you for this suggestion. We would like to stay with our current allocation as this also reflects the protocol of the SR. All four outcomes were primary outcomes at the beginning of this systematic review. We opted to present only the outcomes that had most data available and permitted a meta-analysis. Furthermore, we tried to focus on outcomes that are less likely to have a bias. As hospital admission for a novel, unknown disease is more likely to occur if just for observation. In addition, ICU admission and intubation are expected to be correlated. |
| If hypertension is not considered cardiovascular disease, could you please specify what diseases where included under the umbrella term “cardiovascular disease”. | ‘Cardiovascular disease’ was used by many authors as an own term. In addition we have included comorbidities such as ‘chronic heart disease’ and ‘coronary artery disease’ under this term. In contrast hypertension was stated as an own disease even when cardiovascular disease was mentioned by the authors. We stated as follows in the **Discussion** at **page 14, lines 278 to 279.**  “*With respect to co-morbidities, we identify cardiovascular disease, which includes chronic heart disease and coronary artery disease (OR 3.93), […]*” |

Reviewer – Discussion

| I think there should be a brief paragraph contrasting current findings with those of previous reviews and why the authors think there might be similarities or contrast. | We appreciate this suggestion. We added a brief paragraph in the **Discussion** section at **pages 15 to 16, lines 312 to 317**.  “*In line with previous reviews, we corroborate risk factors such as age, dyspnea, smoking, diabetes, hypertension and cardiovascular disease for mortality.*  *On the one hand we did not find an increased risk for asthma, whereas ‘respiratory diseases’ had an increased OR in other reviews. On the other hand, we identify COPD as a strong risk factor for mortality. This can be explained by an evolving set of data during the pandemic and different grouping of diseases. Nevertheless, leading to the suggestion that lung diseases are associated with an increased risk of a severe course*.” |
| --- | --- |
| Assessing clinical relevance? Does statistically significant result mean automatically clinically relevant? | Thank you for this question. No, statistically significant results do not mean automatically clinically relevant results. We made a statement in the **Methods, Statistical analysis** section explaining this approach at **page 7, lines 130 to 132**.  “*Clinical significance was* determined by *expert consensus with clinicians. A predefined rule (e.g. 10% above normal) across biomarkers or vital parameters is not possible as it is different for every marker and the unit chosen*.” |

Reviewer – Limitation

| Consider mentioning:  - Missing radiological findings as risk factors - When it is mentioned that: “We also assessed the association with hospital and intubation…, but here confounding factors seem to be even more pronounced”. I got confused because confounding factors where not mentioned previously in the text for the other outcomes. This might imply that the author knows what they mean about the confounding factors of the other outcomes, but this is not mentioned in the text. | Thank you for those considerations. We have implemented and explained the limitations accordingly.  Please find an example of the confounding factors and mentioning of missing radiological findings at the **Limitation** section at **page 16 lines 329 to 332 and 335 to 338.**  “*Confounding factors that lead to this conclusion are for example different health care systems across the globe with or without the possibility of self care in less severe cases instead of hospital admission or the change in the approach regarding early intubation from the first wave towards a more conservative approach with novel methods such as ‘awake proning’*.”  “*We have not assessed radiological findings as risk factors as those will likely correlate with clinical signs and symptoms, as well as changes in laboratory parameters. In contrast to other systematic reviews, we did not focus on the course of the disease (e.g. critical, severe), but rather on the outcome. Furthermore, risk factors were assessed for each outcome individually*.” |
| --- | --- |
| I think something like this should be mentioned:  - **The aim is to analyze individual outcomes. However, quality analysis was performed at the study and not the outcome level. This imply that the conclusion of the evidence quality analysis might be valid, but not necessarily so. More granular analysis of the evidence outcome level might yield different results.** It is possible that this is not the case, but I think it is important to mentioned for methodological reasons. | Thank you for this suggestion. We have incorporated your statement at the **Discussion** section as stated above. |

Reviewer – Conclusion

| I think it is a bit overconfident to suggest that the review “confirms” the previous and new indicators as risk factors for covid-19 severity. Please do not take this comment as an attempt to undermine the academic value of the contribution. Both systematic review and meta-analysis are considered the highest quality of evidence in health-related research. However, the review did not evaluate evidence quality at the outcome level. This method is specifically designed to evaluate evidence quality (and not only risk of bias) at the outcome level and use this information to develop clinical guidelines.  I would suggest that the word “confirm” should be replaced by support. | Thank you for this comment. We didn’t want to come across overconfident and followed your suggestion to change the word to “corroborates” in our **Conclusion** section, **page 17, line 352**.  “*Our data on mortality and ICU admission corroborates most of the proposed indicators of clinical outcomes, clarifies the strength of association and highlights additional indicators*.” |
| --- | --- |
